# Prognostic impact of insulin‐like growth factor‐I and its binding proteins, insulin‐like growth factor‐I binding protein‐2 and ‐3, on adverse histopathological features and survival outcomes after radical cystectomy

**DOI:** 10.1111/iju.14869

**Published:** 2022-04-02

**Authors:** Reza Sari Motlagh, Victor M Schuettfort, Keiichiro Mori, Satoshi Katayama, Pawel Rajwa, Abdulmajeed Aydh, Nico C Grossmann, Ekaterina Laukhtina, Benjamin Pradere, Hadi Mostafai, Fahad Quhal, Mohammad Abufaraj, Richard Lee, Pierre I Karakiewicz, Yair Lotan, Eva Comprate, Marco Moschini, Paolo Gontero, Shahrokh F Shariat

**Affiliations:** ^1^ Department of Urology, Comprehensive Cancer Center Medical University of Vienna Vienna Austria; ^2^ Men's Health and Reproductive Health Research Center Shahid Beheshti University of Medical Sciences Tehran Iran; ^3^ Department of Urology University Medical Center Hamburg‐Eppendorf Hamburg Germany; ^4^ Department of Urology The Jikei University School of Medicine Tokyo Japan; ^5^ Department of Urology Okayama University Graduate School of Medicine, Dentistry and Pharmaceutical Sciences Okayama Japan; ^6^ Department of Urology Medical University of Silesia Zabrze Poland; ^7^ Department of Urology King Faisal Medical City Abha Saudi Arabia; ^8^ Department of Urology University Hospital Zurich Zurich Switzerland; ^9^ Institute for Urology and Reproductive Health Sechenov University Moscow Russia; ^10^ Research Center for Evidence Based Medicine Tabriz University of Medical Sciences Tabriz Iran; ^11^ Department of Urology King Fahad Specialist Hospital Dammam Saudi Arabia; ^12^ Department of Special Surgery Jordan University Hospital, The University of Jordan Amman Jordan; ^13^ Department of Urology Weill Cornell Medical College New York New York USA; ^14^ Cancer Prognostics and Health Outcomes Unit University of Montreal Health Center Montreal Quebec Canada; ^15^ Department of Urology University of Texas Southwestern Medical Center Dallas Texas USA; ^16^ Department of Pathology Medical University of Vienna Vienna Austria; ^17^ Unit of Urology/Division of Oncology URI, IRCCS Ospedale San Raffaele Milan Italy; ^18^ Division of Urology, Molinette Hospital University of Torino School of Medicine Torino Italy; ^19^ Department of Urology, Second Faculty of Medicine Charles University Prague Czech Republic; ^20^ Karl Landsteiner Institute of Urology and Andrology Vienna Austria; ^21^ Hourani Center for Applied Scientific Research Al‐Ahliyya Amman University Amman Jordan

**Keywords:** binding proteins, bladder cancer, IGF‐I, insulin‐like growth factor, radical cystectomy, urothelial carcinoma

## Abstract

**Objectives:**

Insulin‐like growth factor‐I and its binding proteins are involved in cancer development, progression, and metastasis. In urothelial carcinoma, the impact of this pathway is still poorly investigated. The present large cohort study aimed to evaluate the association of preoperative circulating levels of insulin‐like growth factor‐I, insulin‐like growth factor‐I binding protein‐2 and ‐3 on outcomes after radical cystectomy.

**Methods:**

A retrospective cohort study of the plasma specimens from 1036 consecutive urothelial carcinoma patients who were treated with radical cystectomy. The primary and secondary outcomes were adverse histopathological features and survival outcomes. Binominal logistic regression and multivariable Cox regression analyses were performed to assess the association of plasma levels of insulin‐like growth factor‐I, insulin‐like growth factor‐I binding protein‐2 and ‐3 with outcomes.

**Results:**

On multivariable analysis adjusting for the effects of preoperative variables, lower insulin‐like growth factor‐I binding protein‐2 levels were associated with an increased risk of lymph node metastasis and (any non‐organ confined disease) any non‐organ confined disease. Insulin‐like growth factor‐I binding protein‐3 levels were also inversely independently associated with lymph node metastasis. Receiver operating characteristic curve analysis showed that the addition of insulin‐like growth factor‐I binding proteins biomarkers to a reference model significantly improved the discriminating ability for the prediction of lymph node metastasis (+10.0%, *P* < 0.001). On multivariable Cox regression models, lower levels of both insulin‐like growth factor‐I binding protein‐2 and ‐3 plasma levels were associated with recurrence‐free survival, cancer‐specific survival, and overall survival. insulin‐like growth factor‐I binding protein‐2 and ‐3 levels and improved the discrimination of a standard reference model for the prediction of recurrence‐free survival, cancer‐specific survival, and overall survival (+4.9%, 4.9%, 2.3%, respectively).

**Conclusions:**

Preoperative insulin‐like growth factor‐I binding protein‐2 and ‐3 are significantly associated with features of biologically and clinically aggressive urothelial carcinoma. These biomarkers improved prognostic urothelial carcinoma models.

**Abbreviations & Acronyms**
AUC: area under the curve
CI: confidence interval
CIS: carcinoma *in situ*
CSS: cancer‐specific survival
DCA: decision curve analysis
HR: hazard ratio
IGFBP: insulin‐like growth factor‐I binding protein
IGF‐I: insulin‐like growth factor‐I
IGF‐IR: insulin‐like growth factor‐I receptor
IQR: interquartile range
LNM: lymph node metastasis
NOCD: non‐organ confined disease
OR: odds ratio
OS: overall survival
RC: radical cystectomy
RFS: recurrence‐free survival
ROC: receiver operating characteristic
SE: standard error
UC: urothelial carcinoma

## Introduction

RC with/without perioperative systemic chemotherapy is the standard treatment of muscle‐invasive non‐metastatic bladder UC; RC is also recommended for very high‐risk non‐muscle‐invasive bladder UC including Bacillus Calmette–Guérin unresponsive disease.^1^, ^2^, ^3^, ^4^ Despite progress, 5 years of CSS and OS of patients treated with RC remain below 60%.^5^ Histopathologic variables such as T stage and lymph node status remain the most important prognostic markers after RC.^6^, ^7^ Previously investigated prognostic and predictive blood‐based biomarkers in patients treated with RC for UC either do not offer a clinically meaningfully discriminative ability and/or lack external validation.^8^, ^9^, ^10^, ^11^, ^12^, ^13^, ^14^, ^15^, ^16^

IGF‐I is produced by liver cells,^17^ tumor cells, and cancer‐associated macrophages.^17^ The IGF‐I signaling pathway, including IGF‐I itself, the circulating levels of IGFBPs, and its tissue receptors (IGF‐IR) have been found to promote tumor cell proliferation, metastasis, and drug resistance.^17^ IGF‐IR has been found to be overexpressed in bladder UC compared to normal urothelium.^18^ Current studies that assessed tissue expression and plasma levels of IGFBP‐2 and ‐3 as a prognostic and/or predictive biomarker in UC patients have been equivocal. We have previously shown that lower preoperative plasma IGFBP‐3 level, in addition to clinical stage and grade, was associated with metastases to regional lymph nodes, bladder cancer progression, and survival.^19^ All studies including ours suffered from small sample size and limited follow‐up.^19^, ^20^, ^21^

To definitively assess the relationship of preoperative plasma levels of IGF‐I, IGFBP‐2, and ‐3 with adverse histopathologic features and survival outcomes in bladder UC, we studied a large consecutive cohort of patients with non‐metastatic bladder UC treated with RC and pelvic lymphadenectomy.

## Methods

### Patients selection

All procedures described in the present study were undertaken with the approval and oversight of the Institutional Review Board for the Protection of Human Subjects of two institutions (IRBs: 1011011386 and 069826900). This study is a retrospective analysis of prospectively collected plasma samples obtained from a consecutive cohort of patients who were treated with RC for non‐metastatic bladder UC at two medical institutions. The extent of lymphadenectomy and choice of urinary diversion were at the surgeon's discretion. Patients with any concomitant secondary malignancy, concomitant upper tract UC, or missing data were excluded. We included any patients who did not receive neoadjuvant chemotherapy. Adjuvant chemotherapy was administered to 167 patients (16.1%) at the clinicians' discretion based on tumor stage and overall health status. No patient received perioperative radiotherapy.

### Biomarker measurements

Sample collection was performed for our biomarker database between 2003 and 2015. Preoperative serum and plasma samples were collected typically on the morning of the day of surgery after an overnight fast. Specimen collection and measurement have been described in detail previously.^22^ Briefly, blood was collected into Vacutainer CPT 8‐ml tubes containing 0.1 mL of 1 m sodium citrate (Becton Dickinson, Franklin Lakes, NJ, USA) and centrifuged at room temperature for 20 min at 1500 × *g*. The top layer corresponding to plasma was decanted using sterile transfer pipettes and immediately frozen and stored at −80°C in polypropylene cryopreservation vials (NalgeNunc, Rochester, NY, USA). For quantitative measurements of IGF‐I, IGFBP‐2, and ‐3 levels, we used quantitative immunoassays (R&D Systems, Minneapolis, MN, USA). Every sample was run in duplicate, and the mean was used. Differences between the two measurements for IGF‐I, IGFBP‐2, and ‐3 were minimal (below 7% for all).

### Pathological review and follow‐up

All surgical specimens were processed according to standard pathological procedures as previously described.^23^ Genitourinary pathologists assigned tumor grades according to the 1973 WHO grading system. The pathologic stage was reassigned according to the 2002 American Joint Committee on Cancer TNM staging system. The presence of concomitant CIS was defined as the presence of CIS in conjunction with another tumor other than CIS. Pelvic lymph nodes were examined grossly, and all lymphoid tissue was submitted for histological examination. The positive soft tissue surgical margin was defined as the presence of tumor at inked areas of soft tissue on the RC specimen except for urethral and/or ureteral margins. Lymphovascular invasion was defined as the unequivocal presence of tumor cells within an endothelium‐lined space without underlying muscular walls.

Clinical and radiological follow‐up was performed following institutional protocols and current guidelines. Routine follow‐up usually included physical examination, radiological imaging, and urine cytology every 3 months for 2 years. Between the second and the fifth years, follow‐up was performed semiannually. Afterward, in most cases, an annual follow‐up was performed. Tumor recurrence was defined as the occurrence of loco‐regional recurrence or distant metastasis on radiological imaging. Cause of death was abstracted from medical charts and/or from death certificates.

### Statistical analysis

Report of categorical variables included frequencies and proportions. Reporting of continuous coded variables focused on medians and IQR. Concerning preoperative plasma levels of IGF‐I, IGFBP‐2, and ‐3, which were routinely treated as continuous variables, group comparisons were performed using the Mann–Whitney *U* tests, Kruskal–Wallis tests, or calculation of Spearman's rank correlation coefficient (*ρ*) and subsequent significance testing, as appropriate. For stratification concerning Kaplan–Meier survival curves, 5‐year survival rates, and patient characteristics, group classification (low *vs* high) was performed using median plasma levels of IGF‐I, IGFBP‐2, and ‐3.

The primary outcome was the adverse histopathological features including LNM, ≥pT3 disease, or any NOCD. Binominal logistic regression analysis was performed for evaluating the association of pretreatment plasma levels of IGF‐I, IGFBP‐2, and ‐3 with LNM, ≥pT3 disease, or any NOCD (i.e., defined as ≥pT3 disease and/or LNM). The AUC of ROC curves was calculated to determine the predictive accuracy of multiple logistic regression models. DeLong's test was used to test for statistical significance between different AUCs. The secondary outcome was survival outcomes including RFS, CSS, and OS. Association between pretreatment IGF‐I, IGFBP‐2, and ‐3 with RFS, CSS, and OS were assessed in univariable and multivariable Cox regression models. Clinical and pathological tumor grade was excluded as a variable for all predictive models since virtually all RC patients had high‐grade bladder UC. Separate Cox regression models that featured either preoperative clinical variables or postoperative histopathological variables were created. The discriminative ability of these models after inclusion of IGF‐1, IGFBP‐2, and/or ‐3 was tested using Harrel's concordance index (C‐index). The additional clinical net benefit of all biomarkers was also evaluated using DCA. Separate reference models, that represented either the preoperative or postoperative setting, were created, to which IGF‐I, IGFBP‐2, and/or ‐3 were added to assess the additive predictive value of each biomarker. All reported *P*‐values were two‐sided, and statistical significance was set at 0.05. All statistical analyses were performed using R Version 3.6.3 (The R Foundation for Statistical Computing, Vienna, Austria).

## Results

### Association with clinicopathologic features

The characteristics of the 1036 patients included in the analysis are displayed in Table S1. There was a significant, yet weak, correlation between plasma levels of IGF‐I and IGFBP‐2 (Spearman's *ρ* = −0.07, *P* = 0.03), IGF‐I and IGFBP‐3 (Spearman's *ρ* = 0.08, *P* = 0.01) and IGFBP‐2 and IGFBP‐3 (Spearman's *ρ* = 0.09, *P* = 0.003). Median plasma levels of IGFBP‐2 and IGFBP‐3 were significantly lower among patients with adverse pathologic features such as lymphovascular invasion, LNM, or positive soft tissue surgical margins (all *P*‐values <0.05, Table S1).

On univariable analyses, pretreatment levels of IGF‐I were not associated with either LNM (OR 1.11, 95% CI 0.9–1.36, *P* = 0.35), pT3/4 disease (OR 1.05, 95% CI 0.87–1.26, *P* = 0.6) or any NOCD (OR 1.02, 95% CI 0.84–1.23, *P* = 0.84). Pretreatment levels of IGFBP‐2 and IGFBP‐3 were inversely associated with LNM (OR 0.58, 95% CI 0.51–0.66, *P* < 0.001 and OR 0.65, 95% CI 0.55–0.77, *P* < 0.001, respectively), pT3/4 disease (OR 0.82, 95% CI 0.74–0.9, *P* < 0.001 and OR 0.85, 95% CI 0.73–0.97, *P* = 0.019, respectively) or any NOCD (OR 0.91, 95% CI 0.83–0.99, *P* = 0.038 and OR 0.84, 95% CI 0.73–0.97, *P* = 0.02, respectively).

**Fig. 1:**
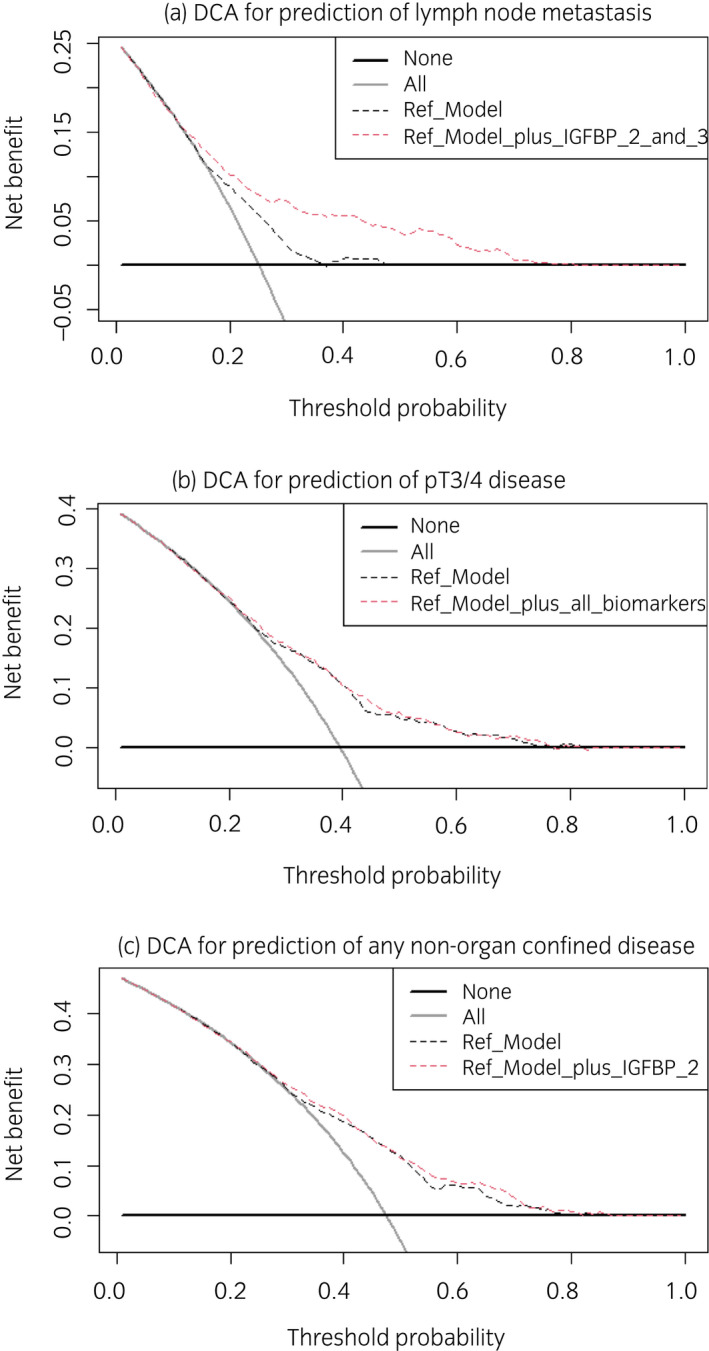
DCA for the net benefit of the preoperative IGF‐1, IGFBP‐2, and IGFBP‐3 plasma levels based on a preoperative reference model (comprising age, sex, and clinical staging) for the prediction of LNM (a), pT3/4 disease (b) or any non‐organ‐confined disease (c) in 1029 patients treated with RC for UC of the bladder. Description: the *x*‐axis is the threshold probabilities. The *y*‐axis measures the net benefit which is calculated by adding the true positives and subtracting the false‐positives. The horizontal line representing the *x*‐axis assumes that no patients experiences recurrence whereas the grey line assumes that all patients will experience recurrence at a specific threshold probability. The dashed black line represents the net‐benefit of a logistic regression reference model which was fitted using sex, age and clinical tumor stage. The dashed red line represents the net‐benefit of the same logistic regression reference models which also include the significant biomarkers from the multivariable analysis as variables. [Colour figure can be viewed at wileyonlinelibrary.com]

On multivariable logistic regression modeling, higher pretreatment levels of IGFBP‐2 and ‐3 were significantly associated with decreased probability of LNM (OR 0.59, 95% CI 0.51–0.68, *P* < 0.001 and OR 0.68, 95% CI 0.57–0.81, *P* < 0.001, respectively; Table 1). There was also a significant association between IGFBP‐2 plasma levels and the risk of any NOCD (OR 0.81, 95% CI 0.73–0.89, *P* < 0.001). ROC curve analysis showed that the addition of IGFBP‐2 and ‐3 biomarkers to a preoperative reference model comprising age, sex, and clinical tumor stage improved the discriminatory ability for prediction of LNM (+10.0%, *P* < 0.001), pT3/4 disease (+1.3%, *P* = 0.024), and any NOCD (+2.4%, *P* = 0.003). When the significant biomarkers from the multivariable models were added in DCA, there was an improvement in net benefit relative to the reference model for prediction of LNM (Fig. 1) within a threshold probability of 20–70%. In our cohort, 569 (55.3%) of all patients fell in this range.

**Table 1:** Preoperative multivariable logistic regression analysis for prediction of lymph node involvement, pT3/4 disease, and any NOCD in 1029 patients treated with RC for UC of the bladder

Characteristic	Lymph node involvement			pT3/4 disease			Any NOCD		
	OR	95% CI	*P*‐value	OR	95% CI	*P*‐value	OR	95% CI	*P*‐value
IGF‐1 levels in μg/cL	1.12	0.89, 1.40	0.3	1.06	0.86, 1.29	0.6	1.07	0.88, 1.31	0.5
IGFBP‐2 levels μg/cL	0.59	0.51, 0.68	<0.001*	0.91	0.82, 1.00	0.052	0.81	0.73, 0.89	<0.001*
IGFBP‐3 levels μg/dL	0.68	0.57, 0.81	<0.001*	0.87	0.75, 1.01	0.076	0.88	0.76, 1.03	0.11
Age	1.00	0.98, 1.01	0.7	1.03	1.01, 1.04	<0.001*	1.02	1.01, 1.03	0.003*
Female sex (ref: male)	1.38	0.96, 1.97	0.077	1.03	0.75, 1.42	0.8	1.13	0.82, 1.55	0.5
Clinical tumor stage (ref: cTa/cTis/cT1)									
cT2	2.57	1.86, 3.57	<0.001*	2.70	2.06, 3.57	<0.001*	3.03	2.32, 3.98	<0.001*
cT3/cT4	3.61	2.00, 6.45	<0.001*	9.01	5.06, 16.8	<0.001*	8.51	4.65, 16.5	<0.001*
AUC of the full model (all variables included)	72.9*			68.8*			70.1*		
AUC of the model without biomarkers levels (reference model)	62.9*			67.5*			67.6*		
Additive value of biomarkers in %; *P* = difference to full model	10% (*P* < 0.001)*			1.3% (*P* = 0.024)*			2.5% (*P* = 0.003)*		

* Statistically significant value.

### Association of plasma levels of IGF‐1, IGFBP‐2, and ‐3 with survival outcomes

**Fig. 2:**
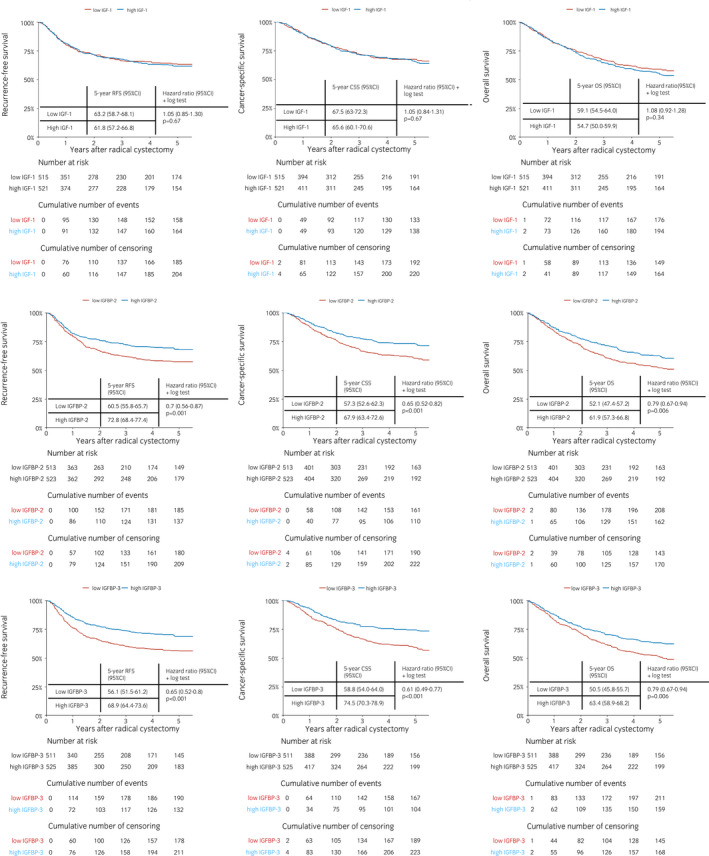
Kaplan–Meier + pairwise log‐rank tests for 5‐year RFS (left column); CSS (middle column); and OS (right column) according to the preoperative median levels of IGF‐1 (first row), IGFBP‐2 (second row), and IGFBP‐3 (third row) in 1036 patients treated with RC for UC of the bladder. [Colour figure can be viewed at wileyonlinelibrary.com]

The median follow‐up of all patients alive was 37.0 months (IQR 14.5–108.5). The 5‐year RFS, CSS, and OS estimates were 62.5% (95% CI 59.2–66.0), 66.6% (95% CI 63.3–70), and 59.6% (95% CI 53.6–60.5), respectively. Stratified by the median pretreatment levels, patients with high IGFBP‐2 and ‐3 had better RFS, CSS, and OS compared to those with IGFBP‐2 and ‐3 below the median (Fig. 2). The 5‐year cumulative number of events on RFS in high IGFBP‐2 *versus* low and high IGFBP‐3 *versus* low were 137 *versus* 185 and 132 *versus* 190, respectively. The 5‐year cumulative number of events on CSS in high IGFBP‐2 *versus* low and high IGFBP‐3 *versus* low were 110 *versus* 161 and 104 *versus* 167, respectively. The 5‐year cumulative number of events on OS in high IGFBP‐2 *versus* low and high IGFBP‐3 *versus* low were 162 *versus* 208 and 159 *versus* 211, respectively.

**Fig. 3:**
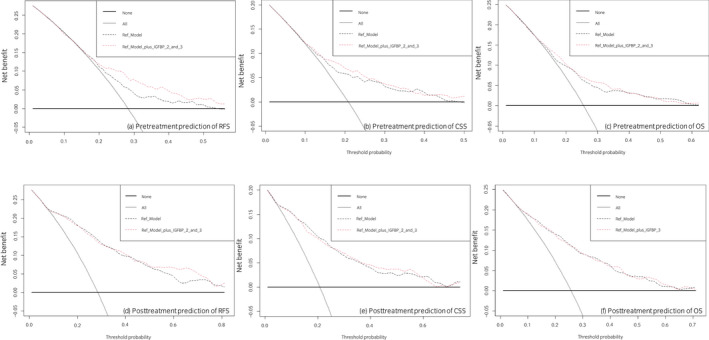
DCA for the additional net benefit of IGFBP‐2 and ‐3 based on a preoperative reference model (upper row: including age, sex, and clinical tumor stage) and a postoperative reference model (lower row: including age, sex, adjuvant chemotherapy, positive surgical margins, pT status, pN status and concomitant CIS on final pathology report) for the prediction of RFS (a + d), CSS (b + e), or OS (c + f) in 1036 patients treated with RC for UC of the bladder. Description: the *x*‐axis is the threshold probabilities. The *y*‐axis measures the net benefit which is calculated by adding the true positives and subtracting the false‐positives. The horizontal line representing the *x*‐axis assumes that no patients experiences recurrence whereas the grey line assumes that all patients will experience recurrence at a specific threshold probability. The dashed black line represents the net‐benefit of a basic Cox regression model which was fitted using above mentioned variables. The dashed red line represents the net‐benefit of the same Cox regression models which also include the preoperative NLR as a variable. [Colour figure can be viewed at wileyonlinelibrary.com]

In preoperative and postoperative multivariable Cox regression models, elevated IGFBP‐2 and ‐3 plasma levels remained independently associated with favorable RFS, CSS, and OS (Table 2; Table S2). The addition of the biomarker levels improved the discriminatory ability of a reference model that included pretreatment clinical variables (sex, age, and clinical stage) for prognosticating of RFS, CSS, and OS (change of C‐Indices <4.9%). The addition of the IGFBP‐2 and ‐3 plasma levels to a reference model based on established posttreatment variables did not improve the model's discriminatory ability (change of C‐Indices <1% for RFS, CSS, and OS). On DCA, there was a small gain in a net benefit for prediction of RFS between disease probabilities of 20–50% and 60–70%, through the addition of IGFBP‐2 and ‐3 plasma levels to reference models based on either established pretreatment or posttreatment variables. The inclusion of the IGFBP‐2 and ‐3 plasma levels did not meaningfully improve the clinical net benefit of separate prognostic models that either included preoperatively or postoperatively available variables across any threshold probability for prediction of CSS or OS (Fig. 3).

**Table 2:** Preoperative multivariable cox regression analysis for prediction of RFS, CSS, and OS in 1029 patients treated with RC for UC of the bladder

Characteristic	RFS			CSS			OS		
	HR	95% CI	*P*‐value	HR	95% CI	*P*‐value	HR	95% CI	*P*‐value
IGF‐1 levels in μg/cL	1.07	0.91, 1.25	0.4	1.09	0.93, 1.28	0.3	1.13	1.00, 1.27	0.054
IGFBP‐2 levels μg/cL	0.78	0.71, 0.86	<0.001*	0.77	0.69, 0.85	<0.001*	0.87	0.81, 0.93	<0.001*
IGFBP‐3 levels μg/dL	0.78	0.68, 0.88	<0.001*	0.74	0.64, 0.84	<0.001*	0.88	0.80, 0.97	0.009*
Age	1.02	1.00, 1.03	0.008*	1.02	1.01, 1.03	<0.001*	1.05	1.04, 1.06	<0.001*
Female sex (ref: male)	1.49	1.16, 1.90	0.002*	1.63	1.26, 2.11	<0.001*	1.32	1.08, 1.60	0.006*
Clinical tumor stage (ref: cTa/cTis/cT1)									
cT2	1.71	1.35, 2.15	<0.001*	1.84	1.44, 2.36	<0.001*	1.66	1.38, 1.98	<0.001*
cT3/cT4	1.97	1.31, 2.96	0.001*	2.21	1.45, 3.38	<0.001*	1.85	1.34, 2.54	<0.001*
C‐Index of the full model (all variables included)	65.4 (SE = 0.016)			68.1 (SE = 0.016)			65.9 (SE = 0.013)		
C‐Index of the model without biomarkers levels (reference model)	60.5 (SE = 0.016)			63.2 (SE = 0.016)			63.6 (SE = 0.013)		
Additive value of the biomarkers in %	4.9%			4.9%			2.3%		

* Statistically significant value.

### Subgroups analyses in patients staged cT1 and cT2 (clinically organ‐confined)

On multivariable logistic regression analyses in patients staged cT2, both IGFBP‐2 and ‐3 were independently associated with LNM, but not pT3/4 disease or any NOCD. The addition of both biomarkers to a predictive reference model (comprising age, sex, and clinical tumor stage) increased the accuracy for the prediction of LNM by a significant margin (+20%, *P* ˂ 0.001). IGFBP‐2 was also independently associated with any NOCD (Table S3). On multivariable Cox regression models, elevated IGFBP‐2 and ‐3 plasma levels were independently associated with favorable RFS and CSS. IGFBP‐2 plasma levels were also independently associated with OS (Table S4). The 5‐year cumulative number of events on RFS in high IGFBP‐2 *versus* low and high IGFBP‐3 *versus* low were 74 *versus* 108 and 74 *versus* 108, respectively. The 5‐year cumulative number of events on CSS in high IGFBP‐2 *versus* low and high IGFBP‐3 *versus* low were 60 *versus* 97 and 62 *versus* 95, respectively. The 5‐year cumulative number of events on OS in high IGFBP‐2 *versus* low was 90 *versus* 120.

The addition of the biomarker levels improved the discriminatory ability of a reference model for prognosticating of RFS, CSS, and OS (change of C‐Index +7%, +7%, and +3%, respectively).

On multivariable logistic regression analyses in patients staged cT1 only, IGFBP‐2 was independently associated with LNM and any NOCD but not with pT3/4 (Table S5). Moreover, the addition of both biomarkers to a predictive model increased the accuracy for the prediction of LNM by a significant margin (+14%, *P* = 0.002). On multivariable Cox regression models, only elevated IGFBP‐3 plasma levels remained independently associated with favorable OS (Table S6). The 5‐year cumulative number of events for OS in high IGFBP‐3 *versus* low was 39 *versus* 53.

## Discussion

The present large cohort study aimed to assess the prognostic role of plasma levels of IGF‐I axis elements (i.e., IGF‐I and IGFBP‐2 and ‐3) in bladder UC. In agreement with our previous study, we failed to find an association between plasma IGF‐I levels and adverse pathologic features or survival outcomes. In contrast, we did find an association of lower pretreatment levels of IGFBP‐2 and ‐3 with adverse pathologic features after RC (i.e., lymphovascular invasion, LNM, and/or positive soft tissue surgical margins). We also found on multivariable analyses that patients with a lower preoperative IGFBPs level had a higher probability of harboring LNM and/or NOCD. In addition, survival analyses demonstrated that in both pretreatment and posttreatment settings, higher levels of both IGFBP‐2 and ‐3 were independently associated with improved RFS, CSS, and OS. While these promising results are consistent with those of previous studies that assessed prognostic/predictive levels of plasma IGFBPs.^19^, ^24^ There are inconsistent results of studies that assessed tissue expression of IGFBPs as prognostic/predictive biomarkers in bladder UC.^20^, ^21^, ^24^, ^25^ Although some studies indicated the tissue expression of IGFBP‐2 but not IGFBP‐3 has a tumor suppressor effect and it is a chemosensitivity marker in UC. Other studies have been shown that the tissue expression levels of IGFBP‐2 in chemoresistant patients were significantly increased, compared with those in the chemosensitive patients.^20^, ^21^, ^25^

Our negative results for the IGF‐I levels might be explained by the fact that 99% of circulating IGF‐I are bound to IGFBPs.^26^ IGFBPs inhibit many IGF‐I actions, including proliferation, survival, migration, differentiation, and nutrient uptake in a wide range of normal and malignant cell types.^17^ Interestingly, IGFBPs have well‐established IGF‐I‐independent actions with specific cell receptors that inhibit cell proliferation, survival, and migration, but may also enhance these processes in a context‐specific manner.^24^, ^27^ Therefore, every element of the IGF‐I axis could play uniquely independent and dependent actions in the pathogenesis of UC. Cohort studies have shown IGF‐IR overexpression in UTUC and bladder UC; moreover, in muscle‐invasive bladder UC, IGF‐IR overexpression was associated with OS and CSS.^18^, ^28^, ^29^

The most important histopathological prognostic variables after RC and LN dissection are tumor stage and LN status.^6^ However, the sensitivity of pretreatment standard imaging, such as computed tomography, for detecting lymph nodes is generally low.^23^ Although biomarkers help improve risk stratification by estimating the probability of treatment failure, their implementation in clinical practice has not been realized due to the absence of adequately designed external validation studies. In addition, to date, there are no reliable pretreatment blood‐based biomarkers to predict lymph node metastases.^30^ To comprehensively evaluate the clinical benefit, we assessed the discriminatory ability of IGFBP‐2 and ‐3 to considerably improve the performance of predictive/prognostic reference models. Our ROC curve analyses showed that the addition of IGFBPs to a reference model improved the ability to predict LNM, NOCD, and pT3/4 disease. Moreover, an additional clinical net benefit for the prediction of LNM occurred upon the addition of IGFBP‐2 and ‐3 in the predictive model, which translated to more accurate prediction for approximately 50% of our patients. Interestingly, in the patient subgroup with only cT2 and only cT1, the addition of both biomarkers to a predictive model increased significantly the accuracy for prediction of LNM by an even greater extent (+20% and +14%, respectively). Thus, pretreatment levels of both biomarkers may help refine patient selection for the delivery of neoadjuvant systemic therapies, even in patients with cT1 UC. However, as the overall accuracy of our models was not high enough to warrant their clinical implementation, further biomarkers, and machine learning are needed to allow their use in clinical settings. Moreover, we found a C‐index improvement in the preoperative setting for RFS, CSS, and OS in all cohorts as well as in the patient subgroup with cT2 only. These findings could also improve the risk stratification for intensified perioperative therapy and follow‐up schedule.

To our knowledge, this study is the first with an acceptable sample size to assess the impact of the IGF‐I axis in bladder UC patients. The main limitations of our study are its retrospective and multicenter design as well as the short median follow‐up of 37 months. It has, however, previously been reported that more than 90% of all patients who experienced disease recurrence would do so within the first 24 months after RC.^31^ Additionally, there are hormonal, metabolic, and nutritional confounding factors that can affect the circulating levels of IGF‐I and its binding proteins thereby may have masked a biologic relationship. Moreover, the bioactivity in tissues of these biomarkers is not correlated with circulating levels.^32^ IGF‐I and its binding protein levels were measured at a single pretreatment time point. While evaluating them over time in response to therapy, recurrence and metastasis will be more informative. Another limitation could be the decision not to include patients treated with neoadjuvant chemotherapy. This was to measure the correlation of these biomarkers with UC biological and clinical behavior in a puristic model with as little as possible confounding factors that alter the natural history of the disease.

In the conclusion, we externally validated that elevated preoperative plasma levels of IGFBP‐2 and ‐3 are associated with RFS, CSS, and OS. In addition, both biomarkers had a significant impact on the prediction of adverse pathologic features such as LNM and advanced tumor stage. Thus, both biomarkers could help identify patients, who may benefit more from a neo‐adjuvant chemo/immune checkpoint therapy. Both biomarkers exhibit the potential effect to improve the discriminatory power of current prognostic models and refine decision‐making to enable individualized patient care.

## Author contributions

Reza Sari Motlagh: Conceptualization; data curation; formal analysis; methodology; writing – original draft. Victor M Schuettfort: Conceptualization; data curation; formal analysis; methodology. Keiichiro Mori: Data curation. Satoshi Katayama: Data curation; formal analysis. Pawel Rajwa: Data curation; supervision. Abdulmajeed Aydh: Data curation; supervision. Nico C Grossmann: Data curation; supervision. Ekaterina Laukhtina: Data curation; supervision. Benjamin Pradere: Data curation. Hadi Mostafaei: Data curation. Fahad Quhal: Data curation. Mohammad Abufaraj: Supervision. Richard Lee: Supervision; writing – review and editing. Pierre I Karakiewicz: Methodology; supervision. Yair Lotan: Supervision. Eva Comprate: Supervision. Marco Moschini: Supervision. Paolo Gontero: Supervision. Shahrokh F Shariat: Conceptualization; methodology; supervision; writing – review and editing.

## Conflict of interest

None declared.

## Approval of the research protocol by an Institutional Reviewer Board

1011011386 and 069826900.

## Informed consent

N/A.

## Registry and the Registration No. of the study/trial

N/A.

## Animal studies

N/A.

## Supporting information


**Table S1.** Association of the pretreatment IGF and its binding proteins with clinicopathologic characteristics in 1036 patients treated RC for UC of the bladder.
**Table S2.** Postreatment multivariable cox regression analysis for prediction of RFS, CSS and OS in 1036 patients treated with RC for UC of the bladder.
**Table S3.** Preoperative multivariable logistic regression analysis for prediction of lymph node involvement, pt3/4 disease and any NOCD in 498 stage t2 patients treated with RC for UC of the bladder.
**Table S4.** Preoperative multivariable cox regression analysis for prediction of RFS, CSS and OS in 498 stage t2 patients treated with RC for UC of the bladder.
**Table S5.** Preoperative multivariable logistic regression analysis for prediction of lymph node involvement, pt3/4 disease and any NOCD in 336 stage t1 patients treated with RC for UC of the bladder.
**Table S6.** Preoperative multivariable cox regression analysis for prediction of RFS, CSS and OS in 336 stage t1 patients treated with RC for UC of the bladder.
