# Supplementary material for: Prognostic impact of insulin‐like growth factor‐I and its binding proteins, insulin‐like growth factor‐I binding protein‐2 and ‐3, on adverse histopathological features and survival outcomes after radical cystectomy
Source: Int J Urol. 2022 Apr 2;29(7):676–83. doi: 10.1111/iju.14869 (PMC9543826; doi:10.1111/iju.14869)
Supplement: Supplementary file 1 — Table S1. Association of the pretreatment IGF and its binding proteins with clinicopathologic characteristics in 1036 patients treated RC for UC of the bladder. Table S2. Postreatment multivariable cox regression analysis for prediction of RFS, CSS and OS in 1036 patients treated with RC for UC of the bladder. Table S3. Preoperative multivariable logistic regression analysis for prediction of lymph node involvement, pt3/4 disease and any NOCD in 498 stage t2 patients treated with RC for UC of the bladder. Table S4. Preoperative multivariable cox regression analysis for prediction of RFS, CSS and OS in 498 stage t2 patients treated with RC for UC of the bladder. Table S5. Preoperative multivariable logistic regression analysis for prediction of lymph node involvement, pt3/4 disease and any NOCD in 336 stage t1 patients treated with RC for UC of the bladder. Table S6. Preoperative multivariable cox regression analysis for prediction of RFS, CSS and OS in 336 stage t1 patients treated with RC for UC of the bladder. [file IJU-29-676-s001.docx]

eTable 1: Association of the pretreatment Insulin-like growth factor (IGF) and its binding proteins with clinicopathologic characteristics in 1,036 patients treated radical cystectomy for urothelial carcinoma of the bladder

eTable 2: Postreatment multivariable cox regression analysis for prediction of recurrence-free survival, cancer-specific survival and overall survival in 1,036 patients treated with radical cystectomy for urothelial carcinoma of the bladder

eTable 3: Preoperative multivariable logistic regression analysis for prediction of lymph node involvement, pt3/4 disease and any non-organ confined disease in 498 stage t2 patients treated with radical cystectomy for urothelial carcinoma of the bladder

eTable 4: Preoperative multivariable cox regression analysis for prediction of recurrence-free survival, cancer-specific survival and overall survival in 498 stage t2 patients treated with radical cystectomy for urothelial carcinoma of the bladder

eTable 5: Preoperative multivariable logistic regression analysis for prediction of lymph node involvement, pt3/4 disease and any non-organ confined disease in 336 stage t1 patients treated with radical cystectomy for urothelial carcinoma of the bladder

eTable 6: Preoperative multivariable cox regression analysis for prediction of recurrence-free survival, cancer-specific survival and overall survival in 336 stage t1 patients treated with radical cystectomy for urothelial carcinoma of the bladder

| **eTable 1: Association of the pretreatment Insulin-like growth factor (IGF) and its binding proteins with clinicopathologic characteristics in 1,036 patients treated radical cystectomy for urothelial carcinoma of the bladder** | | | | | | | | | | |
| --- | --- | --- | --- | --- | --- | --- | --- | --- | --- | --- |
|  | **Overall** | **Stratified** **by** **median** **Insulin-like** **growth** **factor** **I** **(IGF-I)** | | | **Stratified** **by** **median** **Insulin-like** **growth** **factor** **(IGF)** **binding** **protein** **2** | | | **Stratified** **by** **median** **Insulin-like** **growth** **factor** **(IGF)** **binding** **protein** **3** | | |
| *Characteristic* | *N* *=* *1,036* | *low,* *N* *=* *515* | *high,* *N* *=* *521* | *p-value* | *low,* *N* *=* *513* | *high,* *N* *=* *523* | *p-value* | *low,* *N* *=* *511* | *high,* *N* *=* *525* | *p-value* |
| Age | 67 (60, 73) | 67 (61, 73) | 66 (59, 73) | 0.14 | 66 (60, 72) | 67 (60, 73) | 0.5 | 67 (59, 73) | 66 (60, 73) | 0.6 |
| Gender |  |  |  | 0.3 |  |  | 0.4 |  |  | 0.8 |
| male | 814 (79%) | 412 (80%) | 402 (77%) |  | 397 (77%) | 417 (80%) |  | 404 (79%) | 410 (78%) |  |
| female | 222 (21%) | 103 (20%) | 119 (23%) |  | 116 (23%) | 106 (20%) |  | 107 (21%) | 115 (22%) |  |
| Blood transfusion | 268 (26%) | 140 (27%) | 128 (25%) | 0.4 | 130 (25%) | 138 (26%) | 0.8 | **150** **(29%)** | **118** **(22%)** | **0.014** |
| Thrombocytosis | 113 (11%) | 55 (11%) | 58 (11%) | 0.9 | 58 (11%) | 55 (11%) | 0.8 | 53 (10%) | 60 (11%) | 0.7 |
| Hypoalbuminemi a | 145 (14%) | 75 (15%) | 70 (13%) | 0.7 | **84** **(16%)** | **61** **(12%)** | **0.036** | **84** **(16%)** | **61** **(12%)** | **0.032** |
| Clinical tumor grade |  |  |  | >0.9 |  |  | 0.4 |  |  | >0.9 |
| Grade 2 | 6 (0.6%) | 3 (0.6%) | 3 (0.6%) |  | 4 (0.8%) | 2 (0.4%) |  | 3 (0.6%) | 3 (0.6%) |  |
| Grade 3 | 1,022 (99%) | 508 (99%) | 514 (99%) |  | 505 (99%) | 517 (100%) |  | 503 (99%) | 519 (99%) |  |
| Unknown | 8 | 4 | 4 |  | 4 | 4 |  | 5 | 3 |  |
| Clinical tumor stage |  |  |  | 0.4 |  |  | 0.4 |  |  | 0.4 |
| cTa | 23 (2.2%) | 10 (2.0%) | 13 (2.5%) |  | 8 (1.6%) | 15 (2.9%) |  | 10 (2.0%) | 13 (2.5%) |  |
| cTis | 105 (10%) | 44 (8.6%) | 61 (12%) |  | 47 (9.2%) | 58 (11%) |  | 41 (8.1%) | 64 (12%) |  |
| cT1 | 336 (33%) | 163 (32%) | 173 (33%) |  | 166 (33%) | 170 (33%) |  | 168 (33%) | 168 (32%) |  |
| cT2 | 498 (48%) | 261 (51%) | 237 (46%) |  | 250 (49%) | 248 (48%) |  | 252 (50%) | 246 (47%) |  |
| cT3 | 38 (3.7%) | 20 (3.9%) | 18 (3.5%) |  | 20 (3.9%) | 18 (3.5%) |  | 20 (4.0%) | 18 (3.4%) |  |
| cT4 | 29 (2.8%) | 13 (2.5%) | 16 (3.1%) |  | 18 (3.5%) | 11 (2.1%) |  | 15 (3.0%) | 14 (2.7%) |  |
| Unknown | 7 | 4 | 3 |  | 4 | 3 |  | 5 | 2 |  |
| Pathological tumor grade |  |  |  | 0.7 |  |  | >0.9 |  |  | 0.8 |
| Grade 1 | 62 (6.0%) | 34 (6.6%) | 28 (5.4%) |  | 31 (6.0%) | 31 (5.9%) |  | 28 (5.5%) | 34 (6.5%) |  |
| Grade 2 | 11 (1.1%) | 6 (1.2%) | 5 (1.0%) |  | 6 (1.2%) | 5 (1.0%) |  | 5 (1.0%) | 6 (1.1%) |  |
| Grade 3 | 963 (93%) | 475 (92%) | 488 (94%) |  | 476 (93%) | 487 (93%) |  | 478 (94%) | 485 (92%) |  |
| Pathological tumor stage |  |  |  | 0.9 |  |  | 0.2 |  |  | 0.2 |
| pT0 | 62 (6.0%) | 34 (6.6%) | 28 (5.4%) |  | 31 (6.0%) | 31 (5.9%) |  | 28 (5.5%) | 34 (6.5%) |  |
| pTa | 22 (2.1%) | 8 (1.6%) | 14 (2.7%) |  | 10 (1.9%) | 12 (2.3%) |  | 13 (2.5%) | 9 (1.7%) |  |
| pTis | 131 (13%) | 65 (13%) | 66 (13%) |  | 60 (12%) | 71 (14%) |  | 55 (11%) | 76 (14%) |  |
| pT1 | 162 (16%) | 83 (16%) | 79 (15%) |  | 70 (14%) | 92 (18%) |  | 81 (16%) | 81 (15%) |  |
| pT2 | 248 (24%) | 121 (23%) | 127 (24%) |  | 128 (25%) | 120 (23%) |  | 114 (22%) | 134 (26%) |  |
| pT3 | 281 (27%) | 138 (27%) | 143 (27%) |  | 138 (27%) | 143 (27%) |  | 148 (29%) | 133 (25%) |  |
| pT4 | 130 (13%) | 66 (13%) | 64 (12%) |  | 76 (15%) | 54 (10%) |  | 72 (14%) | 58 (11%) |  |
| Positive surgical margins | 95 (9.2%) | 46 (8.9%) | 49 (9.4%) | 0.9 | 54 (11%) | 41 (7.8%) | 0.2 | **62** **(12%)** | **33** **(6.3%)** | **0.002** |
| Lymphovascular invasion | 295 (28%) | 140 (27%) | 155 (30%) | 0.4 | **173** **(34%)** | **122** **(23%)** | **<0.001** | 154 (30%) | 141 (27%) | 0.3 |
| Concomitant Carcinoma in situ | 572 (55%) | 288 (56%) | 284 (55%) | 0.7 | 284 (55%) | 288 (55%) | >0.9 | 284 (56%) | 288 (55%) | 0.9 |
| Lymph node metastasis | 263 (25%) | 130 (25%) | 133 (26%) | >0.9 | **172** **(34%)** | **91** **(17%)** | **<0.001** | **159** **(31%)** | **104** **(20%)** | **<0.001** |
| Adjuvant chemotherapy | 167 (16%) | 95 (18%) | 72 (14%) | 0.052 | 93 (18%) | 74 (14%) | 0.10 | 91 (18%) | 76 (14%) | 0.2 |
| Statistics presented: median (IQR); n (%) | | | | | | | | | | |

| **eTABLE 2: Postreatment multivariable cox regression analysis for prediction of recurrence-free survival, cancer-specific survival and overall survival in 1,036 patients treated with radical cystectomy for urothelial carcinoma of the bladder** | | | | | | | | | |
| --- | --- | --- | --- | --- | --- | --- | --- | --- | --- |
|  | Recurrence-free Survival | | | Cancer-specific survival | | | Overall survival | | |
| Characteristic | HR | 95% CI | p-value | HR | 95% CI | p-value | HR | 95% CI | p-value |
| IGF-1 levels in μg/cL | 1.05 | 0.89, 1.24 | 0.5 | 1.09 | 0.92, 1.29 | 0.3 | 1.10 | 0.97, 1.25 | 0.13 |
| IGFBP-2 levels μg/cL | **0.90** | **0.82,** **0.98** | **0.015** | **0.90** | **0.82,** **0.99** | **0.034** | **0.94** | **0.88,** **1.01** | 0.079 |
| IGFBP-3 levels μg/dL | **0.82** | **0.72,** **0.93** | **0.002** | **0.80** | **0.70,** **0.92** | **0.001** | **0.89** | **0.81,** **0.98** | **0.019** |
| Age | 1.01 | 1.00, 1.02 | 0.2 | 1.02 | 1.00, 1.03 | 0.011 | 1.04 | 1.03, 1.05 | **<0.001** |
| Female sex (Ref: male) | **1.47** | **1.15,** **1.89** | **0.002** | **1.56** | **1.20,** **2.01** | **<0.001** | **1.35** | **1.11,** **1.64** | **0.003** |
| Pathological_tumor_stage (Ref: pT0/pTa/pTis/pT1) |  |  |  |  |  |  |  |  |  |
| pT2 | **1.50** | **1.04,** **2.18** | **0.031** | 1.47 | 0.99, 2.18 | 0.056 | **1.41** | **1.10,** **1.80** | **0.006** |
| pT3/pT4 | **3.26** | **2.30,** **4.62** | **<0.001** | **3.13** | **2.16,** **4.54** | **<0.001** | **2.55** | **1.99,** **3.25** | **<0.001** |
| Positive surgical margins (Ref: no) | **1.38** | **1.01,** **1.89** | **0.044** | **1.46** | **1.06,** **2.02** | **0.022** | 1.09 | 0.82, 1.45 | 0.5 |
| Lymphovascular_invasion (Ref: no) | **1.40** | **1.09,** **1.80** | **0.009** | **1.56** | **1.20,** **2.03** | **<0.001** | 1.22 | 1.00, 1.49 | 0.053 |
| Concomitant Carcinoma in situ (Ref: no) | 1.02 | 0.81, 1.28 | 0.9 | 0.92 | 0.73, 1.17 | 0.5 | 0.99 | 0.83, 1.18 | 0.9 |
| Lymph node involvement (Ref: no) | **2.21** | **1.69,** **2.87** | **<0.001** | **2.24** | **1.69,** **2.95** | **<0.001** | **1.95** | **1.57,** **2.42** | **<0.001** |
| Adjuvant_chemotherapy (Ref: no) | 0.89 | 0.68, 1.18 | 0.4 | 0.96 | 0.72, 1.28 | 0.8 | 0.86 | 0.68, 1.08 | 0.2 |
| **C-Index** **of** **the** **full** **model** **(all** **variables** **included)** | 75.4 (se = 0.013 ) | | | 77.9 (se = 0.012 ) | | | 73.3 (se = 0.011 ) | | |
| **C-Index** **of** **the** **model** **without** **biomarker** **levels** **(reference** **model)** | 75.4 (se = 0.012 ) | | | 77.8 (se = 0.012 ) | | | 73.3 (se = 0.011 ) | | |
| **Additive** **value** **of** **the** **biomarkers** **in** **%** | 0% | | | 0.1% | | | 0% | | |

| **eTABALE 3: Preoperative multivariable logistic regression analysis for prediction of lymph node involvement, pt3/4 disease and any non-organ confined disease in 498 stage T2 patients treated with radical cystectomy for urothelial carcinoma of the bladder** | | | | | | | | | |
| --- | --- | --- | --- | --- | --- | --- | --- | --- | --- |
|  | **Lymph node involvement** | | | **pT3/4 disease** | | | **Any non-organ confined disease** | | |
| *Characteristic* | *OR* | *95% CI* | *p-value* | *OR* | *95% CI* | *p-value* | *OR* | *95% CI* | *p-value* |
| IGF-I levels in μg/cL | 1.13 | 0.84, 1.52 | 0.4 | 1.09 | 0.83, 1.43 | 0.5 | 1.16 | 0.88, 1.54 | 0.3 |
| IGFBP-2 levels μg/cL | 0.60 | 0.50, 0.71 | **<0.001** | 0.91 | 0.80, 1.03 | 0.13 | 0.81 | 0.71, 0.92 | **0.002** |
| IGFBP-3 levels μg/dL | 0.63 | 0.49, 0.80 | **<0.001** | 0.90 | 0.73, 1.11 | 0.3 | 0.87 | 0.70, 1.08 | 0.2 |
| Age | 1.00 | 0.98, 1.02 | 0.9 | 1.03 | 1.01, 1.05 | **0.002** | 1.03 | 1.01, 1.05 | **0.005** |
| Female sex  (ref Male | 1.19 | 0.73, 1.94 | 0.5 | 1.05 | 0.68, 1.64 | 0.8 | 1.12 | 0.71, 1.77 | 0.6 |
| **AUC of the full model (all variables included)** | 0.706 | | | 0.593 | | | 0.619 | | |
| **AUC of the model without biomarkers levels (reference model)** | 0.508 | | | 0.571 | | | 0.570 | | |
| **Additive value of biomarkers in %; p= difference to full model** | 20%, p˂0.001 | | | Not significant | | | 4%, p= 0.03 | | |
| OR = Odds Ratio, CI = Confidence Interval, AUC= Area under the curve | | | | | | | | | |

| **eTABLE 4: Preoperative multivariable cox regression analysis for prediction of recurrence-free survival, cancer-specific survival and overall survival in 498 stage T2 patients treated with radical cystectomy for urothelial carcinoma of the bladder** | | | | | | | | | |
| --- | --- | --- | --- | --- | --- | --- | --- | --- | --- |
|  | **Recurrence-free Survival** | | | **Cancer-specific survival** | | | **Overall survival** | | |
| *Characteristic* | *HR* | *95% CI* | *p-value* | *HR* | *95% CI* | *p-value* | *HR* | *95% CI* | *p-value* |
| IGF-I levels in μg/cL | 1.06 | 0.87, 1.30 | 0.6 | 1.08 | 0.88, 1.33 | 0.5 | 1.13 | 0.96, 1.32 | 0.14 |
| IGFBP-2 levels μg/cL | 0.75 | 0.66, 0.84 | **<0.001** | 0.74 | 0.65, 0.84 | **<0.001** | 0.85 | 0.78, 0.93 | **<0.001** |
| IGFBP-3 levels μg/dL | 0.76 | 0.63, 0.90 | **0.002** | 0.74 | 0.61, 0.89 | **0.001** | 0.95 | 0.82, 1.08 | 0.4 |
| Age | 1.01 | 0.99, 1.03 | 0.2 | 1.01 | 1.00, 1.03 | 0.11 | 1.05 | 1.03, 1.06 | **<0.001** |
| Female sex (ref: male) | 1.46 | 1.04, 2.06 | **0.028** | 1.64 | 1.16, 2.32 | **0.006** | 1.36 | 1.04, 1.77 | **0.025** |
| **C-Index of the full model (all variables included)** | 0.638 | | | 0.649 | | | 0.634 | | |
| **C-Index of the model without biomarkers levels (reference model)** | 0.562 | | | 0.577 | | | 0.609 | | |
| **Additive value of the biomarkers in %** | 7% | | | 7% | | | 3% | | |
| HR = Hazard Ratio, CI = Confidence Interval, se = standard error | | | | | | | | | |

| **eTABLE 5: Preoperative multivariable logistic regression analysis for prediction of lymph node involvement, pt3/4 disease and any non-organ confined disease in 336 stage T1 patients treated with radical cystectomy for urothelial carcinoma of the bladder** | | | | | | | | | |
| --- | --- | --- | --- | --- | --- | --- | --- | --- | --- |
|  | **Lymph node involvement** | | | **pT3/4 disease** | | | **Any non-organ confined disease** | | |
| *Characteristic* | *OR* | *95% CI* | *p-value* | *OR* | *95% CI* | *p-value* | *OR* | *95% CI* | *p-value* |
| IGF-I levels in μg/cL | 0.92 | 0.56, 1.47 | 0.7 | 0.89 | 0.61, 1.28 | 0.5 | 0.86 | 0.60, 1.23 | 0.4 |
| IGFBP-2 levels μg/cL | 0.52 | 0.38, 0.70 | **<0.001** | 0.92 | 0.76, 1.11 | 0.4 | 0.80 | 0.66, 0.96 | **0.021** |
| IGFBP-3 levels μg/dL | 0.83 | 0.60, 1.15 | 0.3 | 1.04 | 0.81, 1.34 | 0.8 | 1.07 | 0.84, 1.37 | 0.6 |
| Age | 0.98 | 0.95, 1.01 | 0.2 | 1.01 | 0.99, 1.04 | 0.3 | 1.00 | 0.98, 1.03 | 0.8 |
| Female sex  (ref Male | 1.37 | 0.66, 2.72 | 0.4 | 0.87 | 0.47, 1.55 | 0.6 | 1.10 | 0.63, 1.89 | 0.7 |
| **AUC of the full model (all variables included)** | 0.694 | | | 0.575 | | | 0.582 | | |
| **AUC of the model without biomarkers levels (reference model)** | 0.550 | | | 0.555 | | | 0.515 | | |
| **Additive value of biomarkers in %; p= difference to full model** | **14%, p= 0.002** | | | Not significant | | | 7%, p= 0.1 | | |
| OR = Odds Ratio, CI = Confidence Interval, AUC= Area under the curve | | | | | | | | | |

| **eTABLE 6: Preoperative multivariable cox regression analysis for prediction of recurrence-free survival, cancer-specific survival and overall survival in 336 stage T1 patients treated with radical cystectomy for urothelial carcinoma of the bladder** | | | | | | | | | |
| --- | --- | --- | --- | --- | --- | --- | --- | --- | --- |
|  | **Recurrence-free Survival** | | | **Cancer-specific survival** | | | **Overall survival** | | |
| *Characteristic* | *HR* | *95% CI* | *p-value* | *HR* | *95% CI* | *p-value* | *HR* | *95% CI* | *p-value* |
| IGF-I levels in μg/cL | 1.01 | 0.72, 1.42 | >0.9 | 1.08 | 0.76, 1.54 | 0.7 | 0.98 | 0.75, 1.28 | 0.9 |
| IGFBP-2 levels μg/cL | 0.89 | 0.73, 1.08 | 0.2 | 0.87 | 0.70, 1.09 | 0.2 | 0.93 | 0.79, 1.10 | 0.4 |
| IGFBP-3 levels μg/dL | 0.94 | 0.74, 1.18 | 0.6 | 0.83 | 0.65, 1.06 | 0.14 | 0.84 | 0.70, 0.99 | **0.039** |
| Age | 1.03 | 1.00, 1.05 | **0.030** | 1.03 | 1.01, 1.06 | **0.018** | 1.05 | 1.03, 1.07 | **<0.001** |
| Female sex (ref: male) | 1.30 | 0.79, 2.14 | 0.3 | 1.49 | 0.89, 2.50 | 0.13 | 1.09 | 0.73, 1.61 | 0.7 |
| **C-Index of the full model (all variables included)** | 0.571 | | | 0.595 | | | 0.611 | | |
| **C-Index of the model without biomarkers levels (reference model)** | 0.595 | | | 0.625 | | | 0.626 | | |
| **Additive value of the biomarkers in %** | 2% | | | 3% | | | 1.5% | | |
| HR = Hazard Ratio, CI = Confidence Interval, se = standard error | | | | | | | | | |
